# The effects of anesthetic drug choice on heart rate variability and echocardiography parameters in cats

**DOI:** 10.1038/s41598-024-51162-z

**Published:** 2024-01-03

**Authors:** Chattida Panprom, Nakrob Pattanapon, Soontaree Petchdee

**Affiliations:** 1 Kasetsart University Veterinary Teaching Hospital Kamphaeng Saen Campus, Faculty of Veterinary Medicine, Kasetsart University, Kamphaeng Saen Campus, Nakorn Pathom, Thailand; 2 Department of Large Animal and Wildlife Clinical Sciences, Faculty of Veterinary Medicine, Kasetsart University, Kamphaeng Saen Campus, Nakorn Pathom, 73140 Thailand

**Keywords:** Cardiac device therapy, Physiology, Cardiology

## Abstract

Heart rate variability (HRV) is one of the assessments of cardiovascular risk during general anesthesia. This study aimed to assess the effects of an anesthetic drug on HRV in cats and to provide information for clinical applications. Twenty-four healthy client-owned cats of various breeds, 12 females and 12 males scheduled for elective surgery, were enrolled in this study. The cats were premedicated and induced with 4 protocols: protocol 1, diazepam (0.3 mg/kg) and propofol (2–4 mg/kg) IV; protocol 2, diazepam (0.3 mg/kg) and alfaxalone (1–3 mg/kg) IV; protocol 3, diazepam (0.3 mg/kg) and ketamine (3–5 mg/kg) IV; and protocol 4, xylazine (1 mg/kg) and tiletamine/zolazepam (Zoletil) (5 mg/kg) IM. The heart rate and HRV of the 24 cats were collected before and at least 1 h after administering the anesthetic drugs. Echocardiography was performed to evaluate heart function. Oscillometric blood pressure monitoring was used to obtain the mean blood pressure. After anesthetic drug administration, higher heart rates were found in cats premedicated and induced with alfaxalone (*p* = 0.045) than in the other protocols. The lowest heart rate (HR) values were found in cats in protocol 4 using xylazine and Zoletil. The HRV low frequency (LF) and high frequency (HF) power ratios increased in all protocols except for cats premedicated and intubated with propofol. The standard deviation of the regular sinus beats (SDNN) was higher in cats premedicated and induced with ketamine than in other anesthetic protocols (*p* = 0.015). An increase in sympathetic activity and reduced HRV is associated with high blood pressure and left atrial dimension. The percentage of fractional shortening (FS) decreased in cats premedicated with ketamine. The results showed that the anesthesia method using diazepam and propofol caused the least disturbance of HRV compared with other anesthesia methods that were used in this study.

## Introduction

Heart rate variability (HRV) has been suggested to be a noninvasive assessment to evaluate autonomic activity^1^. Previous studies have reported the response of heart rate variability to many effects of medications in animals, and it has been used as a predictor of mortality in cardiovascular conditions^2–6^. HRV includes two domains: the time domain and the frequency domain. Time domain parameters are measured from the R-R peak of the ECG, which can be calculated as SDNN (the standard deviation of the normal-to-normal R-R intervals). The frequency domain or spectral analysis is measured by transforming ECG signals into spectral signals. A previous study showed that the progression of cardiac conditions could alter cardiac autonomic activity, which leads to an increase in heart rate and a decrease in HRV^7,8^.

Cardiovascular complications can develop in cats undergoing anesthesia. Anesthesia protocols have been studied to improve outcomes and provide safety for anesthesia in cat patients^9^. Previous studies reported a significant relationship between HRV and hypotension. HRV changes were used to predict hypotension after general anesthesia^10^.

Propofol is a common anesthetic drug that has been used in dogs and cats. However, prolonged recovery in cats^11^ and respiratory depression in dogs^12^ are reported with propofol. Alfaxalone is an anesthetic that can be used to sedate an animal at low doses. Alfaxalone is a sedative with a fast onset of effects that is commonly used for inducing short-term anesthesia in cats. Alfaxalone provides muscle relaxation and a rapid recovery. Previous studies reported that alfaxalone had minimal cardiovascular effects^4,13^. Alfaxalone is relatively safe and especially suitable for patients with heart disease, hypotension, and old age^14^. Ketamine is an effective drug for anesthetic induction in dogs and cats and is presumed to provide effects associated with an increased heart rate and can induce postoperative hyperthermia^15^. A combination of xylazine and Zoletil has been used as an ideal anesthetic agent in many animals, including cats, due to the reversible effects of Xylazine and its inexpensive costs^16^.

The anesthesia drugs will affect cardiac parameters. Echocardiography examination and HRV analysis can provide additional information for the selection of anesthesia in cats. However, the benefits of HRV analysis for anesthesia in cats still need to be investigated. Concerning the various usages of anesthetic agents in cats, no studies have been performed to analyze cardiac autonomic activity using HRV to identify anesthetic agents that provide minimal cardiovascular effects. The objective of this study was to evaluate the effects of various anesthetic agents on cardiac autonomic nervous activity using HRV analysis and to provide information for clinical applications. The results from this study could be used to indicate the presence of health problems, including heart conditions. We hypothesized that the minimal effects of anesthetic agents on the cardiovascular system in cats could be characterized by echocardiography combined with cardiac autonomic activity using heart rate and heart rate variability analysis.

## Materials and methods

### Animals

Twenty-four client-owned cats aged 2.6 ± 0.4 years and weighing 3.4 ± 0.2 kg who underwent the surgical procedure at Kasetsart University Veterinary Teaching Hospital Kamphaeng Saen were enrolled in this study. The study protocol was approved by the Institute Animal Care and Use Committee, Kasetsart University (ACKU-62-VET-059). All methods were carried out in accordance with university guidelines, the approved Animal Care and Use Committee, and the ARRIVE guidelines and regulations. The cat's owner was provided with an informed consent form to approve the surgical procedure.

All cats were housed individually indoors throughout the study period. Cats were fasted 12 h prior to the surgery and were randomly assigned into 4 groups of 4 anesthetic protocols, each of which contained six cats. A 23G ¾ inch intravenous catheter was inserted into the cephalic vein using a restraint with a towel for fluid administration during anesthesia to prevent hypotension and hypovolemia and to maintain perfusion. In case the cat was uncooperative with intravenous injections. The cat will receive a collar, and the staff will help to restrain the cat by transferring the cat from their cage to a squeeze cage for intramuscular injection. Cats in protocol 1 (4 males, 2 females) were premedicated, and anesthesia was induced with diazepam (0.3 mg/kg) and propofol (2–4 mg/kg) administered intravenously. Cats in protocol 2 (2 males, 4 females) underwent anesthesia using diazepam (0.3 mg/kg) and alfaxalone (1–3 mg/kg) administered intravenously. Diazepam (0.3 mg/kg) and ketamine (3–5 mg/kg) were administered intravenously in protocol 3 (3 males, 3 females), and xylazine (1 mg/kg) and tiletamine/zolazepam (Zoletil) (5 mg/kg) were used in protocol 4 administered intramuscularly (3 males, 3 females). The induction of anesthesia was administered intravenously or intramuscularly. The drugs (propofol, alfaxalone, and ketamine) administered intravenously were first given half the dose rapidly (over 5–10 s). Then, an additional amount was titrated intravenously to achieve stage 3 plane 2 of anesthesia. Xylazine and tiletamine/zolazepam (Zoletil) were administered intramuscularly into the quadricep muscles. Then, all cats were maintained under anesthesia for approximately 1 h with 2% isoflurane with an oxygen flow rate of 1 L/min. Non-rebreathing circuits (NRC) are used for anesthesia in our study. The blood pressure was monitored during anesthesia in all cats. The vapor setting can be reduced if the cat has low blood pressure. The settings as low as 0.5–1.0% isoflurane may be sufficient to maintain anesthesia in some cats. The cats in the xylazine and tiletamine/zolazepam (Zoletil) protocol received only oxygen throughout the period of anesthesia. The depth of anesthesia was according to stage 3 plane 2 in all cats, which is characterized by muscular relaxation, loss of response to a nociceptive stimulus, loss of pain sensation, and powers of coordinated movement. All parameters were recorded continuously for further analysis using a blinded assessment. The person who recorded all the parameters during anesthesia and the person who interpreted the results were two different people, and neither of them knew what they were testing.

### Clinical evaluations

**Figure 1:**
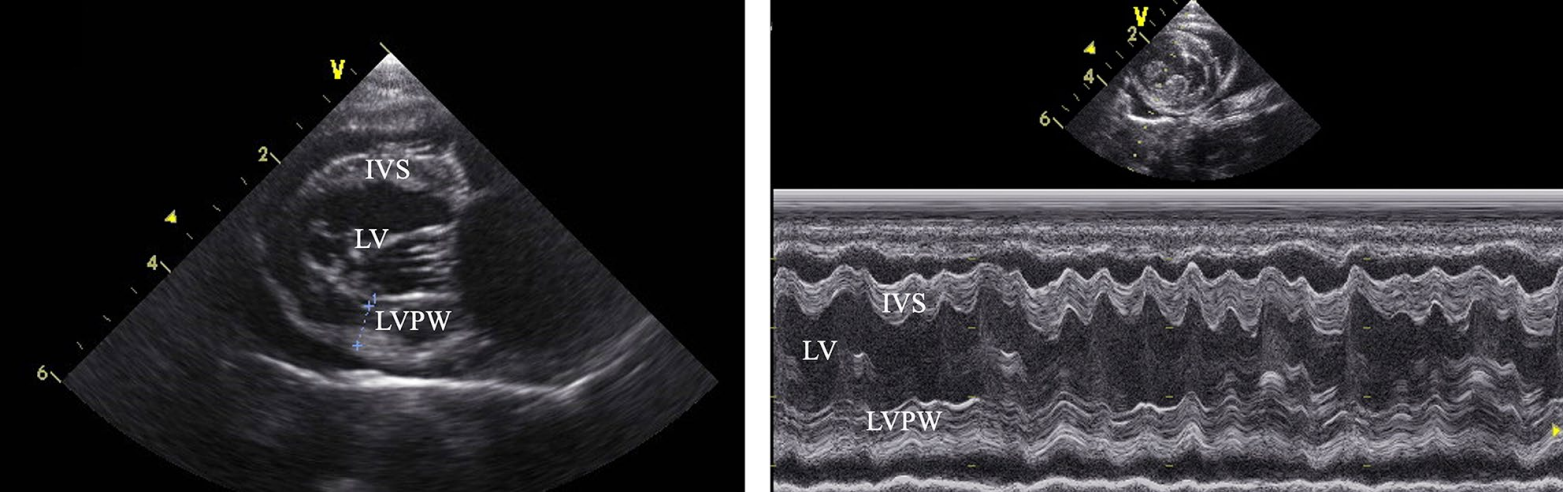
Echocardiographic of a transverse image of heart base (left) and M mode image of the left ventricle (right); *IVS* interventricular septum thickness, *LV* left ventricle, *LVPW* left ventricular proximal wall thickness.

Routine laboratory evaluations were performed, including hematology and serum biochemistry analysis, as shown in Table 1. Cats with abnormal blood profile values were excluded from the study. The blood pressure measurement procedure during anesthesia is performed periodically every five minutes using an automatic noninvasive oscillometer cuff, the cuff width is 30–40% of the circumference of the site where the cuff was applied either forelimb or hindlimb. Electrocardiograms were recorded continuously throughout the experiment to evaluate heart rate and heart rate variability. Transthoracic echocardiography was performed by a standard technique using a vivid 5 s machine with a 6 MHz probe. Parasternal long, short axis, and apical four-chamber views in the right and left parasternal positions were taken. Echocardiographic images were captured and stored for offline analysis using a blinded assessment. Left ventricular wall structure and function were calculated by measuring the images from the two-dimensional plane (Fig. 1).

**Table 1:** A baseline of the blood profiles in four group protocols, protocol 1, diazepam (0.3 mg/kg) and propofol (2–4 mg/kg) IV; protocol 2, diazepam (0.3 mg/kg) and alfaxalone (1–3 mg/kg) IV; protocol 3, diazepam (0.3 mg/kg) and ketamine (3–5 mg/kg) IV; and protocol 4, xylazine (1 mg/kg) and tiletamine/zolazepam (Zoletil) (5 mg/kg) IM.

	Protocol 1 N = 6	Protocol 2 N = 6	Protocol 3 N = 6	Protocol 4 N = 6	Reference range
WBC (× 10^3^/ul)	17.76 ± 1.69	17.58 ± 3.57	20.47 ± 1.56	17.80 ± 1.75	5.5–19
RBC (× 10^6^/ul)	7.83 ± 0.55	7.48 ± 0.75	6.86 ± 0.34	5.34 ± 0.25	5–10
HGB (g/dL)	12 ± 0.85	11 ± 1.03	10 ± 0.44	11 ± 0.31	10–15
HCT (%)	33 ± 2.61	33 ± 2.44	32 ± 1.23	33 ± 1.29	30–45
PLT (× 10^3^/ul)	333 ± 3.3	298 ± 5.31	416 ± 5.19	311 ± 9.50	300–700
PROT (g %)	7.67 ± 0.21	8.20 ± 0.08	7.53 ± 0.21	7.45 ± 0.42	5.8–7.8
BUN (mg%)	21 ± 2.89	26 ± 1.05	20 ± 3.05	18 ± 3.85	15–34
Creatinine (mg%)	1.13 ± 0.10	1.29 ± 0.17	1.21 ± 0.12	1.06 ± 0.59	< 2.0
ALT (IU/L)	76 ± 2.18	47 ± 4.66	43 ± 1.33	29 ± 0.11	28–76

Data are represented as the mean ± SEM, *WBC* White blood cell; *RBC* Red blood cell; *HGB* hemoglobin; *HCT* hematocrit; *PLT* Platelet; *PROT* Total protein; *ALT* Alanine aminotransferase.

### HRV measurements

All cats underwent 3-channel 24-h Holter ECG monitoring (BTL Medical Technologies, Thailand). Noninvasive cardiac autonomic nervous control measurements were performed on uploaded recordings by blinded assessment. Five electrodes were placed on the skin of the thorax with elastic tape to provide ECG recording for the baseline and was continuous for 1 h after administering anesthetic drugs. HRV analysis was performed according to a previously reported study^17^, and the baseline HRV was recorded on the day of surgery before the start of anesthesia and continued until after the operation ended. All animals in this study remained anesthetized for 1 h. The ECGs were recorded before anesthesia, and continuously at least 1 h from the end of the first recording, to ensure the stability of the HRV parameters. The cats received elective surgery, such as orthopedic or soft tissue surgery. Although cats undergo different surgeries, there was no significant difference between the groups regarding the duration of surgery.

Heart rate variability (HRV) was analyzed for the time domain and the frequency domain, and time domain parameters were represented as the standard deviation of the RR interval or SDNN. The frequency domain parameters were measured using fast-Fourier transform analysis in two frequency bands: 0.15–0.5 Hz as a high frequency (HF) and 0.04–0.15 Hz as a low frequency (LF).

### Statistical analysis

All data are shown as the mean ± standard error of the mean (mean ± SEM). In our present study, the standard error of the mean was used to estimate how far the sample mean is likely to be from the population mean. The continuous variables and the differences between anesthetic protocols were analyzed using paired t tests and one-way ANOVA. A correlation matrix was used to represent the pair correlation of all the variables, and correlation coefficients were used to describe the association between variables and to analyze the multiple linear regression models that contained several independent variables. (GraphPad Prism Software version 9.0, USA). A *p* value of 0.05 or less was considered statistically significant.

## Results and discussion

All 24 cats (age 2.6 ± 0.4 years and weighing 3.4 ± 0.2 kg) completed the study without any surgical complications. There were no significant differences among the groups concerning mean age, body weight, or duration of surgery (*p* = 0.9641, *p* = 0.9311, and *p* = 0.9747, respectively) including the sex ratio. The results of blood profiles and cardiac function before the surgical procedure are summarized in Tables 1 and 2, respectively. The blood profiles and echocardiographic parameters in all groups were within normal limits, and no significant differences among the groups were noted. The results from Table 2 show that the average LA internal diameter after anesthesia was increased in protocols 1, 2, and 4. However, in the anesthetic protocol 2, results showed that the LA mean values tended to be more dilated than in the other protocols.

**Table 2:** Echocardiographic parameters before and after surgery in the 4 protocols, protocol 1, diazepam (0.3 mg/kg) and propofol (2–4 mg/kg) IV; protocol 2, diazepam (0.3 mg/kg) and alfaxalone (1–3 mg/kg) IV; protocol 3, diazepam (0.3 mg/kg) and ketamine (3–5 mg/kg) IV; and protocol 4, xylazine (1 mg/kg) and tiletamine/zolazepam (Zoletil) (5 mg/kg) IM.

Parameters		Protocol 1 N = 6	Protocol 2 N = 6	Protocol 3 N = 6	Protocol 4 N = 6	Reference range^26^
IVSd (cm)	Before	0.43 ± 0.02	0.50 ± 0.02	0.50 ± 0.05	0.57 ± 0.05	0.35–0.43
	After	0.43 ± 0.02	0.56 ± 0.04	0.52 ± 0.05	0.53 ± 0.05	
LVIDd (cm)	Before	1.53 ± 0.13	1.34 ± 0.09	1.26 ± 0.20	1.40 ± 0.01	1.43–1.70
	After	1.35 ± 0.14	1.33 ± 0.09	1.42 ± 0.17	1.40 ± 0.01	
LVPWd (cm)	Before	0.43 ± 0.03	0.53 ± 0.04	0.54 ± 0.06	0.47 ± 0.05	0.34–0.43
	After	0.42 ± 0.04	0.53 ± 0.05	0.53 ± 0.04	0.53 ± 0.06	
IVSs (cm)	Before	0.55 ± 0.02	0.59 ± 0.05	0.60 ± 0.06	0.63 ± 0.06	0.55–0.70
	After	0.50 ± 0.03	0.59 ± 0.04	0.55 ± 0.04	0.60 ± 0.07	
LVIDs (cm)	Before	0.95 ± 0.09	0.77 ± 0.04	0.80 ± 0.14	0.80 ± 0.04	0.73–0.98
	After	0.80 ± 0.07	0.90 ± 0.06	1.02 ± 0.14	1.03 ± 0.20	
LVPWs (cm)	Before	0.53 ± 0.02	0.57 ± 0.04	0.56 ± 0.06	0.63 ± 0.02	0.58–0.72
	After	0.50 ± 0.03	0.57 ± 0.04	0.55 ± 0.07	0.60 ± 0.07	
FS (%)	Before	38.0 ± 3.65	42.0 ± 1.31	38.0 ± 2.31	44.6 ± 2.72	39–51
	After	38.3 ± 3.39	33.7 ± 2.16	29.17 ± 1.96	33.6 ± 5.95	
LA (cm)	Before	0.94 ± 0.12	1.02 ± 0.05	1.16 ± 0.09	1.03 ± 0.02	0.96–1.20
	After	1.02 ± 0.13	1.17 ± 0.03	1.10 ± 0.07	1.07 ± 0.02	
LA/Ao Ratio	Before	1.41 ± 0.03	1.57 ± 0.07	1.40 ± 0.06	1.53 ± 0.06	1.06–1.24
	After	1.47 ± 0.03	1.53 ± 0.06	1.46 ± 0.08	1.43 ± 0.06	
IVRT (msec)	Before	60.0 ± 3.39	52.7 ± 1.57	55.0 ± 2.87	51.7 ± 1.18	45–55
	After	62.2 ± 3.24	58.0 ± 4.95	55.3 ± 2.85	70.3 ± 7.04	

Data are represented as the mean ± SEM, *IVSd* diastolic interventricular septum thickness, *IVSs* systolic interventricular septum thickness, *LVIDd* left ventricular end-diastolic diameter, *LVIDs* left ventricular end-systolic diameter, *LVPWd* left ventricular wall diastolic thickness, *LVPWs* left ventricular wall systolic thickness, *FS* left ventricular fractional shortening, *LA* left atrium, *AO* aorta, *LA*/*AO* left atrium diameter per aorta diameter ratio, *IVRT* isovolumic relaxation time.

The vital signs were recorded before during and after anesthesia using the cuff that was connected to a monitor. The monitoring devices measured heart rate, respiratory rate, temperature, pulse oximetry, and blood pressure. The monitor will have alarms that can be set to alert when readings are out of the accepted range. The heart rate decreased after the drug was administered in almost all groups except the protocol 2 group. Cats given protocol 4 had the lowest HR values (90 ± 7 beats/min), and cats given protocol 2 had the highest HR (181 ± 15 beats/min), as shown in Table 3.

**Table 3:** Comparison of heart rate, heart rate variability, and blood pressure after anesthesia in 4 groups of protocols; protocol 1, diazepam (0.3 mg/kg) and propofol (2–4 mg/kg) IV; protocol 2, diazepam (0.3 mg/kg) and alfaxalone (1–3 mg/kg) IV; protocol 3, diazepam (0.3 mg/kg) and ketamine (3–5 mg/kg) IV; and protocol 4, xylazine (1 mg/kg) and tiletamine/zolazepam (Zoletil) (5 mg/kg) IM.

	Protocol 1 N = 6	Protocol 2 N = 6	Protocol 3 N = 6	Protocol 4 N = 6
HR	142 ± 8	181 ± 15	146 ± 18	90 ± 7
SBP	152 ± 7.6	165 ± 12.3	138 ± 7.03	110 ± 10.8
SDNN	244.8 ± 27.2	83.1 ± 15.6	336.6 ± 47.2	221.5 ± 28.8
LF	1.85 ± 0.7	3.39 ± 1.2	2.23 ± 0.9	2.84 ± 1.6
HF	1.62 ± 0.5	2.39 ± 0.7	1.65 ± 0.6	2.74 ± 1.2
LF/HF	0.97 ± 0.1	1.37 ± 0.2	1.38 ± 0.1	0.98 ± 0.2

Data are represented as the mean ± SEM, *HR* heart rate, *SBP* systolic blood pressure, *SDNN* standard deviation of the R–R intervals, *LF* low frequency, *HF* high-frequency, *LF*/*HF* low frequency per high-frequency ratio.

**Figure 2:**
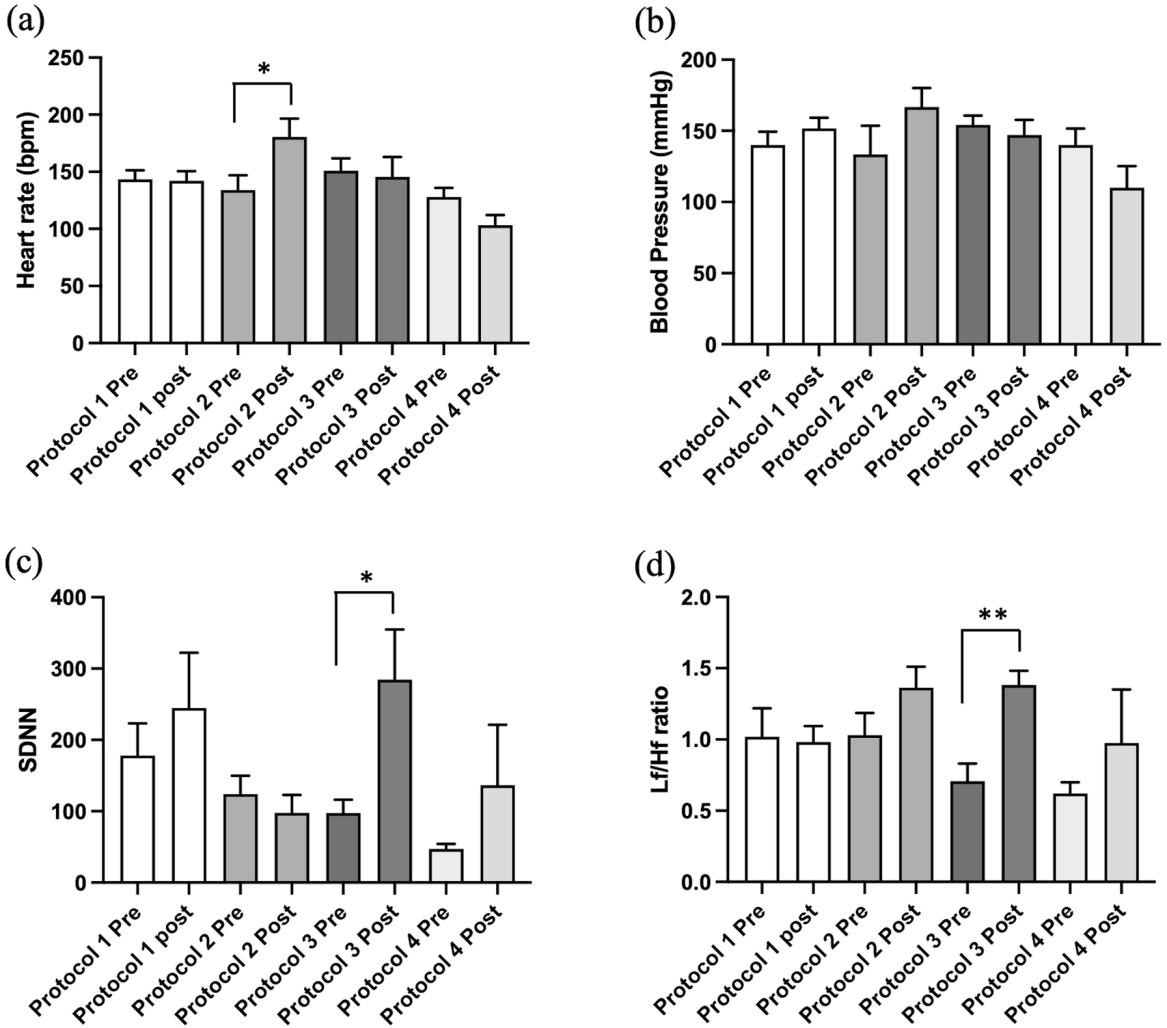
Effect of anesthetic drugs on heart rate (**a**), blood pressure (**b**), and heart rate variability (**c**) standard deviation of the R–R intervals (SDNN), (**d**) low frequency per high-frequency ratio (LF/HF). Values are expressed as the mean ± standard error of the mean (SEM). * Indicates *p* < 0.05, ** indicates *p* < 0.01. The *p value* summary for blood pressure was not significant in all protocols (*p value* summary = 0.1442, Protocol 1 pre vs. post, *p value* = 0.3572, Protocol 2 pre vs. post, *p value* = 0.2415, Protocol 3 pre vs. post, *p value* = 0.5754, Protocol 4 pre vs. post, *p value* = 0.1922).

It has been demonstrated that hypotension during anesthesia may worsen patient outcomes^17^. The systolic blood pressure was decreased in the protocol 4 group (*p* = 0.03). However, those values were within normal limits. The standard deviation of the R-R interval (SDNN) increased after anesthetic drug administration in almost all groups except for protocol 2 (alfaxalone), indicating a reduction in parasympathetic tone, which was related to the significant increase in the heart rate in this group (Fig. 2). LF was used as a marker of sympathetic activity^8^. The highest values of LF power were in the group of protocol 2, which indicated vagal inhibition. The results from this study showed that diazepam and alfaxalone (protocol 2) produced an increase in heart rate and influenced sympathetic activity. Sympathetic activity is increased and correlated with the left atrium (LA) dimension.

Left atrium enlargement is accepted as a result of pressure or volume overload and reflects systolic and diastolic dysfunction^18^. The relationship between LA enlargement and increased sympathetic activity has been shown in cats with anesthetic protocol 2. An enlargement of the LA and increased filling pressure is thought to indirectly stimulate the cardiac autonomic system, including sympathetic and vagal nerve activities^19^. A previous study reported the correlation between left atrium dimensions and the risk of cardiovascular events. Assessment of LA size function, pulmonary veins, and transmitral inflow are used to assess LV diastolic function^20^.

**Figure 3:**
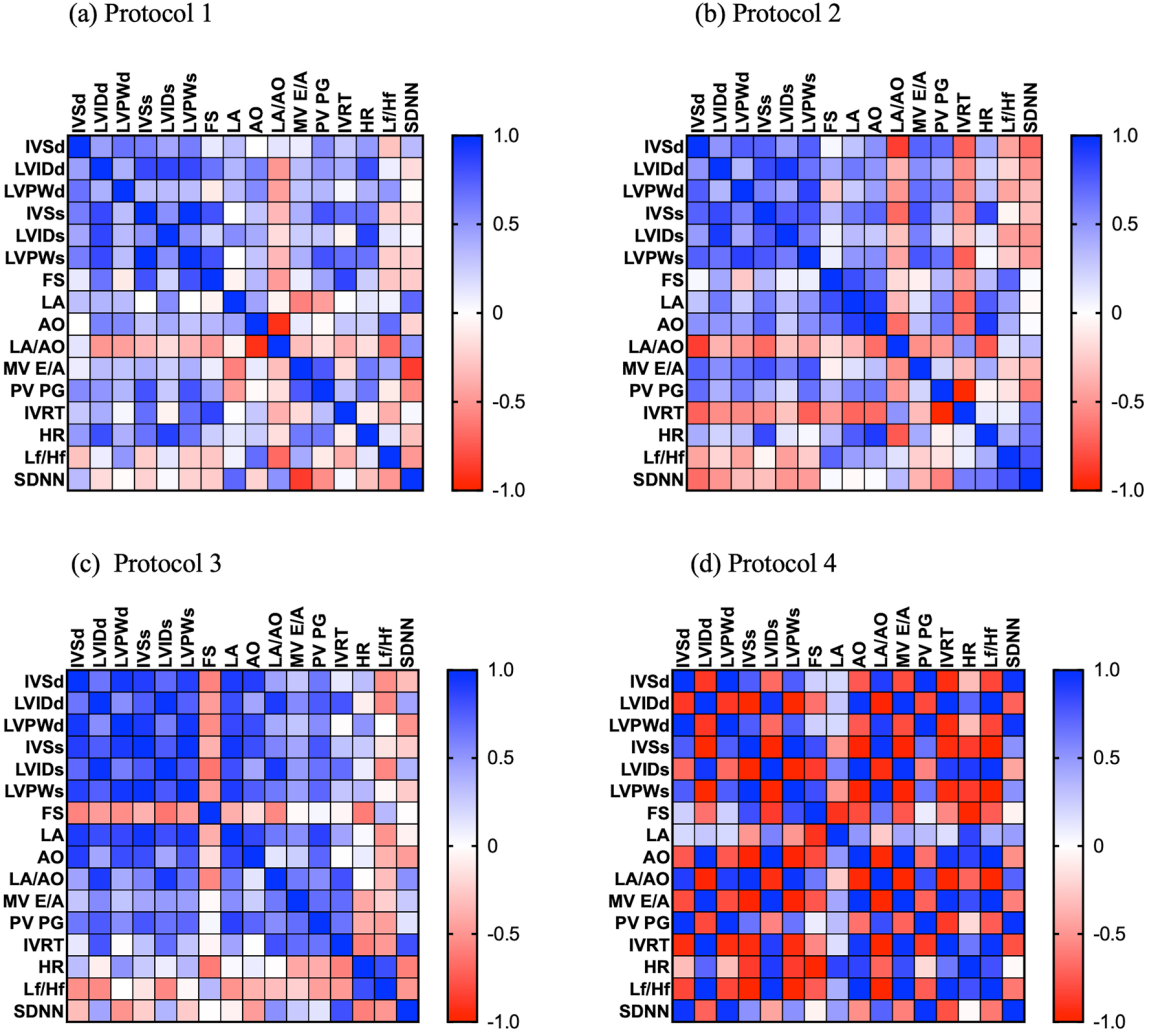
Correlation matrix of all variables used in the cross-lagged models of cats in all anesthetic protocols. Protocol 1 (**a**), protocol 2 (**b**), protocol 3 (**c**), and protocol 4 (**d**). The color bar represents the correlation coefficients from − 1 (red) to + 1 (blue). Blue squares represent significant positive correlations. Red squares represent significant negative correlations.

The present study showed the correlation between echocardiography variables related to HR and HRV. The studies of cats given 4 anesthetic protocols demonstrated a correlation between left ventricle contractile function and LA and LV dimensions. In the present study, anesthetic protocol groups 3 and 4 had an increase in LV internal diameter when compared to anesthetic protocols 1 and 2. In cats given protocol 4, LA exhibited a strong negative correlation with fractional shortening and a weak positive correlation with the isovolumetric relaxation time (IVRT) (Fig. 3). An increase in LA diameter leads to an increase in left atrial volume, which promotes increased diastolic flow velocity in early diastole, thereby shortening the isovolumetric relaxation period. The results were similar to our expectations, and IVRT did not change with an increase in LA diameter. In addition, these left atrial diameter parameters exhibited weak positive correlations with the HRV time and frequency domains in all anesthetic protocols except anesthetic protocol 3 (Fig. 3). Previous study also reported that Ketamine combined with alfaxalone and methadone enhanced sedation. Alfaxalone and butorphanol also had minimal effects on echocardiography^21^. These results suggested that the left atrium dimension might be used as an indicator to adjust the rate of fluid maintenance when using alfaxalone as an anesthesia drug to prevent pressure‒volume overload and to preserve diastolic function as well as cardiovascular outcomes.

In addition, a prolonged isovolumic relaxation time (IVRT) is an early indicator of left ventricular diastolic dysfunction. Previous studies showed a negative correlation of HR with E/A and IVRT^22^. IVRT showed the highest increase in anesthetic protocol 4 using xylazine combined with Zoletil, but it was preserved in anesthetic protocol 3 using ketamine. The side effects of xylazine include bradycardia, depression of the cardiopulmonary system, and cardiac arrhythmias. Tiletamine, which is in the same class as ketamine, has been reported to have a cardiopulmonary similar impact as Ketamine^23^. In this study, ketamine has cardiovascular stimulating effects such as increasing BP, HR, and cardiac output. A combination of ketamine and xylazine provides muscle relaxation, analgesic effects, and unwanted effects of both agents. A combination of ketamine and xylazine may be useful as an anesthetic drug in cats with ventricular dysfunction due to hypertrophic cardiomyopathy. However, IVRT should be used to evaluate diastolic function in combination with other echocardiography variables, such as the transmitral inflow profile^20^.

A previous study demonstrated that low LF⁄HF was an independent predictor of long-term mortality, cardiac events, and the risk of adverse events^24^. Decreased LF⁄HF can be used as a predictor for hypotension or bradycardia. HRV may be used as a suitable tool to identify patients at high risk of a hemodynamic event, such as canine monocytic Ehrlichiosis with an imbalance in the activity of the autonomic nervous system from severe anemia and hypoalbuminemia^17,25^.

In our study, the cats in protocol 1 had slightly reduced values of the LF/HF ratio and decreased percentage changes in heart rate variability when compared with other protocols. These results suggested that diazepam and propofol might preserve the cardiac autonomic activity balance better than the other anesthetic protocols. A marked HRV result for the time domain and frequency domain variation was observed in cats with anesthetic protocol 3 (*p* < 0.05, *p* < 0.01). These results indicated that the ability of the ECG Holter device to measure the time and frequency domains in cats was affected by the choice of anesthetic protocol. However, an insufficient number of cats in each group could affect the statistical analysis, which is a limitation of this study. Furthermore, only one set of ECGs was recorded for 1 h, and recording for only a short period (1 h) during a surgical protocol may affect the results. HRV analysis in anesthetized cats still requires further investigation to ensure its validity for use in future applications.

## Conclusions

HRV can be used to identify cats at high risk of hematological events and to indicate the need for cardiac monitoring after surgery. The findings of this study could be used to indicate the presence of health problems, including heart conditions. Propofol could be used as a premedication and during intubation in cats since it had less effect on cardiovascular function than the other protocols. Results from this study suggested that diazepam (0.3 mg/kg) combined with propofol (2–4 mg/kg) may contribute to the maintenance of cardiovascular stability in anesthetized cats.

### Supplementary Information

Supplementary Information.

## Data Availability

All data generated or analyzed during this study are included in this published article, and the raw data are available in the supplemental files. The datasets used and/or analyzed during the current study are available from the corresponding author upon reasonable request.
